# Anatomical Injection Guidelines for Glabellar Frown Lines Based on Ultrasonographic Evaluation

**DOI:** 10.3390/toxins14010017

**Published:** 2021-12-25

**Authors:** Soo-Bin Kim, Hyoung-Moon Kim, Haeryun Ahn, You-Jin Choi, Kyung-Seok Hu, Wook Oh, Hee-Jin Kim

**Affiliations:** 1 Department of Oral Biology, Division in Anatomy and Developmental Biology, Human Identification Research Institute, BK21 FOUR Project, Yonsei University College of Dentistry, 50-1 Yonsei-ro, Seodaemun-gu, Seoul 03722, Korea; tnqls1128@yuhs.ac (S.-B.K.); haeryonsei@yuhs.ac (H.A.); cyj7797@yuhs.ac (Y.-J.C.); hks318@yuhs.ac (K.-S.H.); 2 Maylin Clinic, Gyeonggi 10391, Korea; drmac12@me.com; 3 Maylin Clinic, Seoul 07335, Korea; feelclinic@naver.com; 4 Department of Materials Science & Engineering, College of Engineering, Yonsei University Seoul, Seoul 03722, Korea

**Keywords:** frowning patterns, glabellar frown lines, ultrasonography, botulinum neurotoxin, injection guideline

## Abstract

When botulinum neurotoxin (BoNT) is injected to treat glabellar frown lines, the corrugator supercilia muscle (CSM) and procerus muscles are the main targets. Although there have been many studies on the treatment of glabellar frown lines, no study has confirmed the dynamic movement under ultrasonography (US). This study examined and evaluated dynamic muscle movements under US, thereby providing more effective BoNT injection guidelines for glabellar frowning. Glabellar frowning was categorized as either Type A or B. Type A is the general frowning pattern in which vertical wrinkles are made by contracting the CSM and procerus muscles (81%, *n* = 13). On US images, the procerus muscle thickens and the bilateral CSMs contract. Type B is an upward frowning pattern demonstrating upward elevation of vertical wrinkles due to hyperactive contraction of the frontalis muscle during frowning (19%, *n* = 3). On US images, the hypoechoic frontalis muscle thickens, forming horizontal forehead lines. After BoNT injection into the CSM and frontalis muscle but not the procerus muscle, Type B patterns showed improvements in the vertical crease and horizontal forehead line. Both types showed improvement in glabellar frown lines after conventional injection, but the horizontal forehead line did not improve in Type B. Type B wrinkles improved after additional injections into the frontalis muscle. This study provided novel anatomical findings related to the injection of glabellar frown lines with BoNT. Preliminary analysis and optimized procedures using US will enable more effective and safer injections.

## 1. Introduction

Glabellar frown lines are generally created by the frontalis, the procerus, and the corrugator supercilii muscles (CSM), and are not only esthetically unattractive but also may result in the individual appearing older than they are or giving a negative facial expression [1]. The CSM has two distinct belly components and depresses the medial eyebrow, forming vertical lines on the glabella. The procerus muscle originating from the nasal bone is intermingled with the frontalis muscle at the insertion point, and this muscle forms a horizontal or curved line on the radix below the glabella by pulling down the medial side of the eyebrow [2]. Based on the anatomical features contributing to glabellar frown lines, the general botulinum neurotoxin (BoNT) injection procedure has been performed in the CSM and procerus muscles [2,3].

There are individual differences in facial expression due to facial muscle variations and the degree of muscle contraction. It is essential to evaluate dynamic anatomical changes in facial muscles [4]. In addition, patient evaluation-based customized treatments are very helpful in meeting the demands of patients achieving optimal results [5]. Meanwhile, the use of ultrasonography (US) has many advantages, in that clinicians obtain accurate and satisfactory results by providing static and dynamic US images to confirm the anatomical changes of muscles in real time [6]. Therefore, US can be used as an essential diagnostic tool during minimally invasive procedures to solve individual anatomical characteristics.

One crucial concept that can aid in the prevention of patient dissatisfaction after BoNT injection, but is often overlooked by injectors, is the understanding of the individual discrepancies of the target muscle’s action during frowning. Knowledge of the muscle territory and dynamics is critical for achieving optimal results when injecting BoNT treatment [5,7]. The aim of this study was to examine and evaluate dynamic muscle movements in the US, thereby providing more effective BoNT injection guidelines for glabellar frowning.

## 2. Results

### 2.1. US-Based Analysis of the Glabellar Frowning Patterns

**Figure 1:**
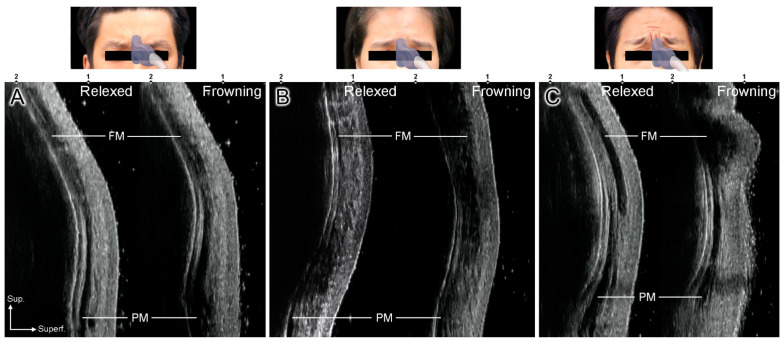
Sagittal ultrasonography (US) images showing the glabellar frowning patterns. (**A**) US images of Type A, (**B**) mild pattern of Type B, and (**C**) severe pattern of Type B. Fm, frontalis muscle; Pm, procerus muscle.

Glabellar frowning was categorized into two patterns according to US. Type A is a general frowning pattern in which vertical wrinkles are formed by the contraction of the CSM and procerus muscles (81%, *n* = 13). On the US images, the procerus muscle thickens as a hypoechoic image and the bilateral CSMs contract (Figure 1A). Type B is an upward frowning pattern demonstrating upward elevation of the vertical wrinkles due to hyperactive contraction of the frontalis muscle during frowning (19%, *n* = 3). On US images, the hypoechoic frontalis muscle is thickened, forming horizontal forehead lines (Figure 1B,C).

Type B was classified into mild and severe subtypes according to the degree of frontalis muscle contraction. In severe Type B, the horizontal forehead lines are prominent because of the hyperactive frontalis muscle (Figure 1C). Mild Type B shows unclear horizontal forehead lines, but definite contraction of the frontalis muscle under US (Figure 1B). Two different glabellar frowning patterns were clearly observed in the sagittal US image compared to the transverse image, and there were no differences between sexes.

### 2.2. Clinical Application

**Figure 2:**
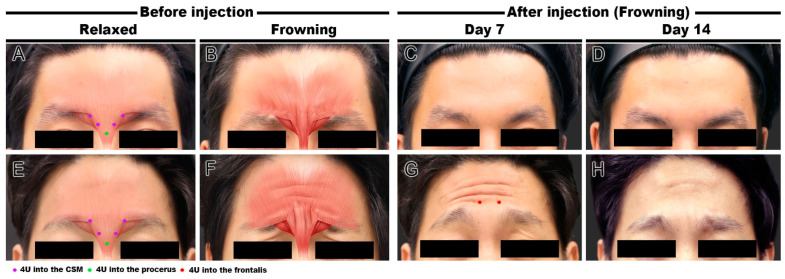
Clinical facial photographs of the conventional injection technique for glabellar frown lines. Type A frowning ((**A**,**B**) and Type B frowning (**E**,**F**)) before botulinum neurotoxin (BoNT) injection. Improvement of glabellar frown lines on day 7 ((**C**,**G**) and day 14 (**D**,**H**)) after injection. Additional two points of BoNT injection into the frontalis muscle on day 7 in Type B frowning (**G**) and final improvement of horizontal forehead lines on day 14 of primary BoNT injection.

**Figure 3:**
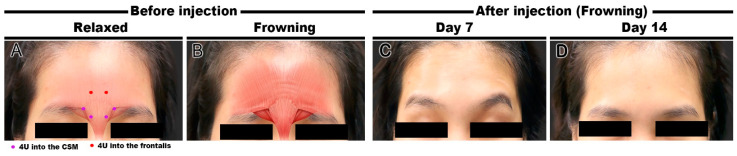
Clinical facial photographs of the modified injection technique for Type B frowning. Before injection (**A**,**B**) and improvement of glabellar frown lines on days 7 (**C**) and 14 (**D**) of injection.

BoNT (Liztox, Humedix Inc., Bundang, Gyeonggi-do, South Korea) was injected into three volunteers to improve glabellar frown lines. After diluting BoNT in 0.9% NaCl solution to a concentration of 100 U/2.5 mL, 0.1 mL (4 U) was injected into each site using a 30 G needle. Five injection points were used for the Type A volunteers, including two points in each CSM and one point in the procerus muscle by the conventional injection technique (Figure 2A). One Type B volunteer was injected using a conventional injection only (Figure 2E), and the other was injected into the CSM and frontalis muscle but not the procerus muscle (Figure 3A). Volunteers were evaluated on days 7 and 14 after BoNT injection.

#### 2.2.1. Cases 1 and 2 (Conventional Injection)

A 26-year-old man and a 36-year-old man both complained of a negative impression due to glabellar frown lines. Clinically, the 26-year-old man showed only vertical lines when frowning (Figure 2A,B), whereas the 36-year-old man displayed horizontal forehead lines due to hyperactive contraction of the frontalis muscle and vertical lines during glabellar frowning (Figure 2E,F). Based on the US evaluation, these clinical symptoms were confirmed and classified as Types A and B, respectively. Conventional BoNT injections were administered to both patients. On day 7 of injection, the glabellar vertical lines disappeared when frowning, and they were well-maintained without other side effects on day 14 (Figure 2C,D). Before BoNT injection in the Type A volunteers, the Merz score was 0 points in glabellar lines at rest, and 4 points in glabellar lines dynamic. Both glabellar lines at rest and glabellar lines dynamic were evaluated as 0 points at day 7 and day 14 after BoNT injection.

In contrast, in the 36-year-old man (Type B), the vertical lines at the glabella disappeared after 7 days but the horizontal forehead lines did not improve (Figure 2G). To improve the forehead horizontal lines, an additional two points of 8 U of BoNT injection was injected into the frontalis muscle above the glabella. On day 7 after the additional injections (day 14 after the primary injections), the horizontal wrinkles had greatly improved (Figure 2H). In the case of the Type B volunteers, the Merz score was 0 points in glabellar lines at rest, 1 point in forehead lines at rest, and 4 points in both glabellar and forehead lines dynamic before BoNT injection. On day 7 after BoNT injection, glabellar lines at rest and dynamic scores were both 0 points. Forehead lines at rest and dynamic scores did not change at all, so that an additional BoNT injection into the frontalis was performed on day 7. Consequently, the Merz scores for forehead lines at rest and forehead lines dynamic were 0 points and 2 points, respectively. According to the patient satisfaction evaluation, the Type A volunteer was satisfied with the BoNT treatment and the Type B volunteer showed a high satisfaction (i.e., very satisfied). In both cases, there were no side effects, such as infection, swelling, or headache.

#### 2.2.2. Case 3 (Modified Injection)

A 32-year-old woman presented with an unnatural cynical impression when frowning. A preliminary clinical photograph showed an upward elevation of the bilateral medial eyebrow margins, followed by slight frontalis muscle contraction (Figure 3A,B). The patient was diagnosed with Type B frowning according to US, and BoNT injections were performed using the improved technique with additional injections into the frontalis muscle above the glabella without injection into the procerus muscle. On day 7 after the injection, complementary contractions of the lateral frontalis muscle (Mephisto phenomenon) were confirmed despite the general improvement of the glabellar wrinkles during frowning (Figure 3C). Although the patient complained of a temporary headache during the week after the injection, the mild side effect disappeared spontaneously. Finally, it was confirmed that horizontal forehead lines disappeared when frowning with the immobilization of the medial frontalis muscle on day 14 of injection (Figure 3D). For this Type B volunteer, the Merz score before BoNT injection was 0 points in both glabellar lines and forehead lines at rest, 1 point in glabellar lines dynamic, and 2 points in forehead lines dynamic. On day 7 after BoNT injection, forehead lines dynamic was measured at 1 point, while three other Merz scores were measured at 0 points. In addition, on day 14 after BoNT injection, all Merz scores of the Type B volunteer were rated as 0 points. This volunteer responded as “satisfied” in the patient satisfaction questionnaire.

## 3. Discussion

There are many differences in muscle volume and intensity during facial animation, and it is necessary to confirm the degree and position of muscle movement under the various facial expressions of the individuals. Despite many studies on the efficacy and safety of BoNT injection procedure for the correction of glabellar frown lines [5,8,9,10], there have been no studies suggesting more effective injection guidelines based on the dynamic anatomical changes of the facial muscle under US. Contrary to previous publications, we provided crucial clinical guidelines to achieve satisfactory outcomes by understanding and evaluating individual anatomical characteristics using US images.

The United States Food and Drug Administration (FDA) approved the treatment of glabellar frown lines using BoNT in 2002 [11], and it is currently the most popular non-invasive treatment [12]. Glabellar frown lines are produced by the fibers of the transverse belly of the CSM attached to the frontalis muscle and the superolateral orbital part of the orbicularis oculi muscle. The transverse belly muscle forms narrow vertical wrinkles in the glabellar region. The procerus muscle is cross-locked with the frontalis muscle fibers, and the muscle forms a horizontal or curved line on the radix below the glabella by pulling down the medial side of the eyebrow. An aged look is created by the transverse belly muscle and frontalis muscle, forming a wrinkle [2]. Therefore, it is recommended to use two injection points into each CSM and one injection point into the procerus muscle [10,13,14] for glabellar frown line treatment.

Various clinical injection guidelines have been proposed to date. Most studies considered the glabellar frown lines to function similarly in the majority of individuals, with only differences related to gender, age, ethnicity, sun exposure, and physical activity [10]. To achieve satisfactory clinical outcomes, it is necessary to understand the complex functional anatomy of the forehead. Furthermore, the physiological conditions of the patients, such as age, sex, and degree of muscle contraction, should be considered using various diagnostic methods [4,5]. Clinically, patients are asked to make various facial expressions to monitor habitual patient-specific facial expressions, and clinicians can understand muscle volume and movements using US. 

In the present study, we classified the frowning patterns into two types based on sagittal images from the US. Contrary to Type A (the general frowning pattern), Type B shows elevation of the medial eyebrow accompanied by horizontal wrinkles due to excessive contraction of the frontalis muscle as well as vertical wrinkles on the glabella. This classification is clinically important because there is a common view that Type B (upward frowning pattern) not only gives the person a nasty appearance or cynical impression, but also brings bad fortune to individuals in Asian countries [15].

We also confirmed the contribution of the procerus muscle during Type B glabellar frowning. From our results, the procerus muscle was not observed at the midline, but appeared to be divided into two lateral portions in the Type B frowning pattern (19%). Lee et al. reported that the procerus muscle in the glabellar region was not clearly detected in 20.3% (type II) [16]. In our study, 56% of the volunteers had procerus muscle type II, and all were females except one volunteer. Therefore, a type II procerus muscle may be related to the Type B glabellar frown line. However, this study had too small of a sample size to confirm the significance of the Type B glabellar frown line and sex with a type II procerus muscle. Therefore, further studies need to be conducted with more cases to analyze such a relationship. 

The use of US for minimally invasive procedures is increasing [17,18,19,20]. As already revealed in many facial US studies, the different echogenicity of the muscle and intramuscular tendon structures can be clearly demonstrated during dynamic movements [21]. Therefore, it is recommended that the most contracted region of the muscle, shown as a hypoechoic US image, could be selected as the target injection point rather than the conventional 5-point blind injection technique. However, in the case of the improved injection technique on the Type B frowning pattern, the volunteer complained of a mild headache and Mephisto phenomenon for 7 days after the injection, which is thought to be caused by the lateral frontalis muscle for the eyebrow elevation and frowning by force. These mild side effects only lasted a week and disappeared spontaneously [22,23,24].

Delicate and various facial expressions are accomplished by the simultaneous activity and interactions of many facial expression muscles [25]. US-based evaluation provides information about the dynamic movement of facial muscles and the clinical application of BoNT. Therefore, US allows clinicians to understand muscle movement and achieve optimal results by considering the relationship with the surrounding muscles. Furthermore, detailed anatomical information about the facial muscles is crucial for optimizing the effectiveness of BoNT treatment. In conclusion, this study provided novel anatomical findings related to the injection of glabellar frown lines with BoNT. Preliminary analysis and optimized procedures using US will enable more effective and safer injections.

## 4. Materials and Methods

**Figure 4:**
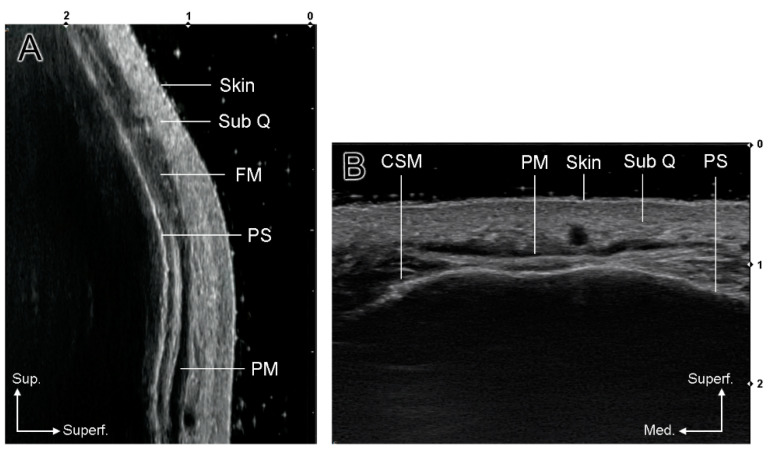
US images of the glabellar region ((**A**), longitudinal view; (**B**), transverse view). (SubQ, subcutaneous layer; FM, frontalis muscle; PM, procerus muscle; CSM, corrugator supercilii muscle; PS, periosteum).

US images of 16 Korean volunteers (9 men and 7 women with a mean age of 28.7 years) were obtained and categorized into two types of glabellar frown lines. Patients were placed in a semi-supine position for US examination. US images of the glabellar region were obtained using a real-time two-dimensional B-mode US device with a high-frequency (18 MHz) linear transducer (Sonimage HS1, KONICA MINOLTA, Tokyo, Japan). All volunteers were asked to relax and make a frowning or angry expression to obtain static and dynamic US images (Figure 2A,B,E,F and Figure 3A,B). US scanning was performed transversely or longitudinally to confirm the muscle dynamics at the glabella. The glabella was defined as the most protruding midline point between the eyebrows on both sides. In the transverse view, movement of the procerus muscle and CSM was identified during frowning (Figure 4B). In the longitudinal view, the position and movement of the procerus and frontalis muscles were observed (Figure 4A). 

The Merz scale was used to evaluate the clinical improvement of glabellar frown lines [26]. The Merz scale is a 5-point scale (0–4 points), with 0 indicating “no line” and 4 indicating “very severe lines”. At all visits, standardized photographs of the treatment site were taken at maximum frown and at rest. A blinded independent investigator completed this evaluation using standardized photographs before BoNT injection, on day 7, and on day 14 after BoNT injection. In addition, the patient satisfaction was assessed according to the “very satisfied”, “satisfied”, “disappointed”, and “very disappointed” scales with a self-report questionnaire at the last visit. The exclusion criteria for this study included pregnancy, history of drug allergy, other serious medical conditions, or surgical or non-surgical treatment of the facial area (including BoNT injection) within the previous 6 months. All procedures performed in this study were approved by the Institutional Review Board of the Yonsei University College of Dentistry (IRB identification code: No. 2-2017-0023; date of approval: 22 June 2017). All subjects received a sufficient explanation of the study purpose and protocols, and they were free to withdraw from the treatment and research at any time.

## Data Availability

The data presented in this study are available on request from the corresponding author.
